# Novel Core–Shell Aerogel Formulation for Drug Delivery Based on Alginate and Konjac Glucomannan: Rational Design Using Artificial Intelligence Tools

**DOI:** 10.3390/polym17141919

**Published:** 2025-07-11

**Authors:** Carlos Illanes-Bordomás, Mariana Landin, Carlos A. García-González

**Affiliations:** AerogelsLab, I+D Farma Group (GI-1645), Department of Pharmacology, Pharmacy and Pharmaceutical Technology, iMATUS and Health Research Institute of Santiago de Compostela (IDIS), Universidade de Santiago de Compostela, E-15782 Santiago de Compostela, Spain; carlosjavier.illanes@rai.usc.es

**Keywords:** aerogels, porous particles, konjac glucomannan, alginate, supercritical CO_2_, artificial intelligence tools, coated particles

## Abstract

This study explores novel alginate–konjac glucomannan core–shell aerogel particles for drug delivery systems fabricated via air-assisted coaxial prilling. A systematic approach is needed for the optimization of this method due to the numerous processing variables involved. This study investigated the influence of six variables: alginate and konjac glucomannan concentrations, compressed airflow, liquid pump pressures, and nozzle configuration. A hybrid software using Artificial Neural Networks and genetic algorithms was used to model and optimize the hydrogel formation, achieving a 100% desirable solution. The optimal formulation identified resulted in particles displaying a log-normal size distribution (*R*^2^ = 0.967) with an average diameter of 1.57 mm. Supercritical CO_2_ drying yielded aerogels with macropores and mesopores and a high specific surface area (201 ± 10 m^2^/g). The loading of vancomycin hydrochloride (Van) or a dexamethasone base (DX) into the aerogel cores during the process was tested. The aerogels exhibited appropriate structural characteristics, and both drugs showed burst release profiles with ca. 80% release within 10 min for DX and medium-dependent release for Van. This study demonstrates the feasibility of producing konjac aerogel particles for delivery systems and the high potential of AI-driven optimization methods, highlighting the need for coating modifications to achieve the desired release profiles.

## 1. Introduction

Bioaerogels are nanostructured and highly porous materials made from natural polymers (polysaccharides or proteins) with a high specific surface area (>100 m^2^/g) and porosity (>95%). Bioaerogels hold significant promise for drug delivery systems [1,2] and can be synthesized via sol–gel processes using a wide variety of polysaccharides, which are Generally Recognized As Safe (GRAS) materials. These aerogels offer advantages like enhanced drug loading, bioavailability, and the ability to stabilize drugs in an amorphous state [3,4]. Despite the versatility of polysaccharides in aerogel production, the potential of many of them, such as konjac glucomannan (KGM), a material with unique properties, remains largely unexplored [5].

Prilling, a scalable technique for producing spherical aerogel particles, enables tunable particle sizes (0.5–4.0 mm) controlled via dropping through nozzles assisted with varied external mechanisms, such as mechanical vibrations, compressed air, or jet-cutting [6,7,8,9,10]. Numerous studies have explored the prilling technique to obtain aerogels for targeted drug release, often using post-prilling coatings. As an example, alginate–pectin aerogels crosslinked with ions (Ca^2+^, Zn^2+^, Sr^2+^) and loaded with diclofenac prevented drug release in Simulated Gastric Fluid (SGF, pH 1.2) but released it immediately in PBS medium (pH 6.8) [7].

Coating technologies have been proposed to prevent immediate release in simulated intestinal fluids. For example, ibuprofen-loaded alginate aerogels with enteric coating, produced by a combination of prilling with supercritical drying and supercritical impregnation followed by a coating process in a Wurster fluidized bed, retained the drug in acidic conditions for 15 min [8]. Similarly, cellulose aerogels loaded with vanillin via supercritical impregnation and coated with shellac resin using a spouted bed extended 50% vanillin release for 90 min, compared to 15 min for uncoated aerogels [9]. Multi-layered alginate aerogels loaded with nicotinic acid also used coatings to extend drug retention at 37 °C and pH 6.5 [10].

Given that aerogel coating technologies are effective, the direct production of core–shell aerogels through prilling emerges as an advantageous integrated approach that has been minimally investigated so far. Core–shell aerogels with a pectin core and an alginate coating for wound healing purposes were developed using vibration-assisted prilling [11]. The preparation involved dissolving alginate and pectin in water, prilling the resulting solutions through a coaxial nozzle into a CaCl_2_ crosslinking ethanolic bath, and drying with supercritical CO_2_. The resulting particles were ca. 3.25 mm in size, had up to 87% drug loading, and achieved sustained release for 48 h. In another example, a core–shell aerogel system loaded with insulin was produced for colonic insulin delivery using air-assisted prilling [12]. The aerogels were obtained from alginate prilled into a chitosan–CaCl_2_ bath, followed by solvent exchange and supercritical CO_2_ drying. These aerogels demonstrated acid stability, improved insulin encapsulation (up to 50%), and reduced premature drug release.

Prilling through the coaxial nozzle technique enables the production of core–shell aerogels with innovative material combinations and tailored functionalities. Moreover, it is a scalable and optimized approach that addresses a significant research gap. However, this technique involves numerous processing variables, making it challenging to predict or analyze the influence of each one on the final formulation characteristics. To overcome this issue, previous studies have employed Design of Experiments (DoE) using two-level fractional factorial designs or multivariable regression analyses. However, these methods either provide limited predictive power or require extensive datasets from DoE [13,14]. While response surface analysis derived from these methods is valuable, its complexity, cost, and time requirements increase with the number of process variables [15,16].

To address these challenges and further enhance formulation design, Artificial Intelligence (AI) tools have been successfully employed in the development and optimization of several dosage forms obtained through complex processes with numerous variables [15,17,18]. Among AI techniques, Artificial Neural Networks (ANNs) stand out for their ability to predict output properties based on different input conditions. Genetic algorithms (GAs), inspired by biological evolution, can optimize formulations by iteratively selecting variable combinations to yield the best performing formulations. Fuzzy logic systems allow for the classification of various results using linguistic rules, which favours the understanding of the processes. These AI tools offer an excellent arsenal for the design of therapeutic systems [19,20,21]. By integrating AI with DoE and other analytical techniques, researchers can accelerate formulation development, reduce costs, and create more effective and targeted drug delivery systems. For example, AI algorithms can be used to predict optimal composition and operation conditions to obtain core–shell aerogel particles based on the desired physical properties and drug release patterns, considering the numerous variables involved in the prilling process [22].

This study aims to design and optimize polysaccharide core–shell aerogel particles composed of an alginate core and a KGM shell, produced through a coaxial nozzle prilling process followed by supercritical CO_2_ drying. KGM, a natural polysaccharide extracted from *Amorphophallus konjac,* possesses remarkable properties, including excellent water-holding capacity, high viscosity, and strong gel-forming ability. It is biocompatible, biodegradable specifically by colonic flora, and non-toxic [23,24,25]. Aerogels using KGM have not previously been reported. It is hypothesized that incorporating KGM into the particle shell will modify drug release profiles for both highly water soluble (Van) and poorly water soluble (DX) drugs, providing novel functionalities to these aerogels as oral dosage forms. The prilling process will be modelled and optimized using AI techniques, considering key parameters as nozzle parameter, flow rates, and pressures to obtain particles with the desired properties.

## 2. Materials and Methods

### 2.1. Materials

Alginic acid sodium salt (G/M ratio of 70/30, Mw 403 kDa) was obtained from Sigma Aldrich (Irvine, UK). High-viscosity konjac glucomannan (Propol A^®^, average molecular weight 200–2000 kDa) was supplied by Shimizu Chemicals (Kihara, Hiroshima, Japan). Dexamethasone base (DX, C_22_H_29_FO_5_, 99.9% purity) and vancomycin hydrochloride (Van, C_66_H_75_Cl_2_N_9_O_24_∙HCl, 94.3% purity) were purchased from Fagron (Nazareth, Belgium) and Guinama (Valencia, Spain), respectively. Absolute ethanol (EtOH absolute), sodium hydroxide (NaOH, 99% purity), and acetonitrile (ACN, HPLC grade) were supplied by VWR Chemicals (Fontenay-sous-Bois, France). Calcium chloride anhydrous (CaCl_2_, MW = 110.98 g/mol) was purchased from Scharlab (Barcelona, Spain), and glacial acetic acid (CH_3_COOH, 99.5% purity) was supplied by Analema-Vorquimica S.L. (Vigo, Spain). Ultrapure nitrogen (N_2_, >99% purity) was provided by Praxair (Madrid, Spain) and used for adsorption–desorption textural analysis. Carbon dioxide (CO_2_, 99.8% purity) from Nippon Gases (Madrid, Spain) was employed as the supercritical fluid for the gel drying process. For the preparation of the release medium, hydrochloric acid (HCl, 37% purity) from Scharlau (Barcelona, Spain), sodium chloride (NaCl, 99.9% purity) from LabKem (Barcelona, Spain), potassium chloride (KCl, 99.9% purity) and potassium dihydrogen phosphate (KH_2_PO_4_, >99.5% purity) from Panreac (Barcelona, Spain), and sodium phosphate dibasic anhydrous (Na_2_PO_4_, pure grade) from Carlo Erba Reagents (Val-de-Reuil, France) were used. Ultrapure water (resistivity > 18 MΩ·cm; Milli-Q^®^ plus, Millipore Ibérica, Madrid, Spain) was used throughout all the experiments.

### 2.2. Feed Solution Preparation

Alginate solutions (0.75–1.25% *w/v*) ([*Alg*] in Table 1) were prepared in Milli-Q^®^ water, magnetically stirred (500 rpm, 24 h), and loaded with the drug (Van or DX, 10 mg/mL) when required. In certain cases, ultramarine deep colourant (Bellas Artes Levante, Alicante, Spain) was added for shell thickness/centering analysis.

KGM solutions (0.6–0.7% *w/v*) (Table 1) were prepared in 0.1 M NaOH and stirred for 24 h to facilitate deacetylation and promote subsequent crosslinking in ethanol (non-solvent-induced phase separation, NIPS). Glacial acetic acid was then added to achieve a final concentration of 1% *v/v*, and the mixture was stirred for 3 min to adjust the pH. After a 30 min settling period, the KGM solution was centrifuged (Centrifuge 5810 R, Eppendorf AG, Hamburg, Germany) at 5000 rpm for 8 min to remove insoluble components.

### 2.3. Preparation of Core–Shell Alcogels

**Figure 1:**
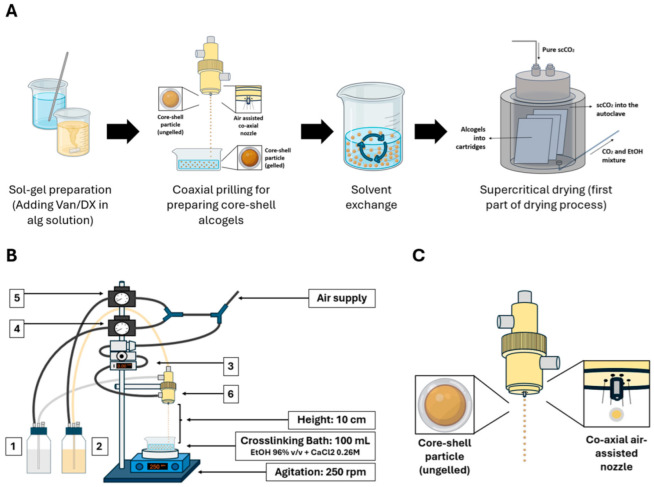
Core–shell aerogel preparation and prilling setup. (**A**) Workflow of core–shell synthesis: gel precursor solution preparation, coaxial prilling and crosslinking, solvent exchange, and supercritical drying (for detailed procedure cf. Section 2.6). (**B**) Prilling setup and experimental variables studied: 1. KGM concentration, 2. Alg concentration, 3. airflow rate, 4. outer solution pressure, 5. inner solution pressure, 6. nozzle configuration. (**C**) Scheme of the coaxial air-assisted prilling nozzle setup. Figures were created using PowerPoint and Biorender.

Core–shell alcogel particles were prepared using a coaxial nozzle prilling system (Nisco Encapsulator Var J1, Zurich, Switzerland) (Figure 1) under different experimental conditions (Table 1). Alginate ([Alg], 0.75–1.25% *w/v*) and KGM ([KGM], 0.6–0.7% *w/v*) solutions were fed to the inner/outer nozzles from pressurized flasks (100 mL). Three Ø_outer/inner_ nozzle configurations (NC) were evaluated: (A) 0.5/0.15 mm; (B) 0.8/0.15 mm, and (C) 0.8/0.35 mm. Droplets fell from a drop height of 10 cm and were crosslinked in 96% ethanol bath with 0.26 M CaCl_2_ under stirring at 250 rpm. Atomization airflow was in the 1.75–2.65 L/min range, with alginate (P_inner_) and KGM (P_outer_) flask pressures set at 0.2–1.0 bar and 0.4–1.2 bar ranges, respectively. Particles were aged (5 min), filtered (mesh size < 1 mm), and transferred to absolute ethanol. Variable ranges for the experimental design (Figure 1) were established by preliminary tests aimed to define the feasible operation region (Table S1).

A reduced experimental design was established using DataForm^®^ v.3.1 software (Intelligensys Ltd., Sale, UK). A balanced design method for six variables was established: (i) KGM concentration ([KGM], % *w/v*), (ii) Alg concentration ([Alg], % *w/v*), (iii) nozzle airflow rate (Airflow, L/min), (iv) inner nozzle flask pressure (P_inner_, bar), (v) outer nozzle flask pressure (P_outer_, bar), and (vi) nozzle configuration. Airflow rate, P_inner_, P_outer_, and nozzle configuration were studied at three levels, while [Alg] and [KGM] were studied at two levels. A minimum of four patterns were included for each variable, resulting in an experimental design comprising 24 formulations produced under different process conditions (Table 1).

### 2.4. Alcogels Characterization

Alcogel particle images were taken using an inverted optical microscope (Olympus IX51, EP5O camera, Olympus, Tokyo, Japan). Particle size (Feret Diameter, mm) and circularity (scale: 0–1) were determined from at least 75 particles per formulation using an automatized method combining ImageJ^®^ v.1.54f software (National Institutes of Health, Bethesda, MD, USA) with the “Trainable Weka Segmentation”, plugin available in Fiji^®^ v.1.54f software (National Institutes of Health, Bethesda, MD, USA) [26]. Coating thickness (mm) was estimated calculating the average thickness of 50 particles by measuring the widest and narrowest part of each coating. To categorize the quality of the blue-dyed alginate core within the particles, the following scoring was used: 0 (no core), 1 (multiple cores), 2 (filament-like core), 3 (uncoated core), 4 (off-centre spherical core), and 5 (centred spherical core).

Finally, prilling capability was scored on severity during batch preparation: 0 (the prilling process fails each few seconds due to the blockage of one or both nozzle sections), 1 (sometimes the prilling flow rate is reduced during the process), and 2 (the prilling flow rate is constant during all process).

### 2.5. Modelling and Optimization of Alcogel Formulations Through Artificial Intelligence Techniques

Composition, operation conditions (Table 1), and measured characteristics for 24 alcogel batches (Table 2) were compiled into a database and modelled using FormRules^®^ v4.03 (Intelligensys Ltd., Sale, UK) and INForm^®^ v5.01 (Intelligensys Ltd., Sale, UK). FormRules^®^, a neuro-fuzzy logic (NFL) technology, generates models that identify input–output patterns and present them as simple, interpretable rules to explain formulation characteristics based on process variables, thereby favouring a deeper understanding of the processes and facilitating knowledge extraction from complex relationships, which is a significant advantage for the rational design and optimization of drug delivery systems [27].

For modelling, six inputs were introduced: nozzle configuration (NC), KGM concentration ([KGM] % *w/v*), alginate concentration ([Alg], % *w/v*), inner and outer nozzle flask pressures (P_inner_, P_outer_, bar), and airflow rate (L/min). Five outputs were introduced: prilling capability score, Feret diameter (mm), circularity, centred core score, and coating thickness (mm).

FormRules^®^ training parameters included a ridge regression factor of 10^−6^, set densities of 2 and 3, a maximum of 2 inputs per submodel, and 15 nodes per input (node adaptation enabled). Among the model selection criteria available in FormRules^®^, Structural Risk Minimization (C1 > 0.75) was chosen, prioritizing high predictability and rule interpretability [20,28].

INForm^®^ is a hybrid technology that integrates ANN and GA tools. It effectively models the relationship between inputs and outputs, identifying the optimal combination of variables to achieve the desired properties of formulations [27,28]. The dataset was randomly split into training data (80%) and testing data (20%) to be modelled using INForm^®^. The software was configured with a momentum of 0.8, a learning rate of 0.7, and a target MS error of 0.0001 over 1000 iterations. A random seed of 10,000 ensured reproducibility, and the Smart Stop feature was enabled to fine-tune the training process. The network architecture included six inputs and one hidden layer with two nodes, using an asymmetric sigmoid transfer function.

The selection of network architecture can be performed by the user via a trial-and-error methodology, exploring various configurations. For this study, the chosen architecture was the software’s default recommendation, informed by the number of inputs and outputs. The output layer used either a linear or asymmetric sigmoid transfer type, with training optimized through the RPROP algorithm.

The quality of the models obtained by FormRules^®^ and INForm^®^ was evaluated by their predictability and accuracy. Predictability was evaluated using the coefficient of determination (*R*^2^) of the correlation between the experimental and predicted parameters for the model, expressed as percentage, as shown in Equation (1). Values of *R*^2^ over 70% indicate reasonable predictabilities [20,27]. Accuracy was assessed through ANOVA, which compares the experimental parameters with the predicted ones. The absence of significant differences indicates a good model performance of the model.

(1) $$R^{2}=\left.\left[\;1-\frac{\;\sum_{i=1}^{n}{\left(y_{i}-{y^{\prime }}_{i}\;\right)}^{2}}{\sum_{i=1}^{n}{\left(y_{i}-{y^{\prime \prime }}_{i}\;\right)}^{2}}\right.\right]\times 100\%$$

where *y_i_* is the actual value in the dataset, *y*′*_i_* is the value calculated by the model, and *y*″*_i_* is the mean of the dependent variable.

Desirability functions were designed to optimize core–shell alcogel production and characteristics. The objective included minimizing nozzle blockage (drop capability score < 1), achieving particles between 2 and 3 mm in size, high circularity (>0.7), a centred core (score > 4), and a coating thickness higher than 0.2 mm.

INForm^®^ optimization used the following default parameters: number of populations of 1, 100 iterations, population size of 100 individuals, replacement rate 50%, mutation standard deviation 0.1, and random seed 1.

Model validation involved the experimental preparation of the optimized formulation and the subsequent comparison of the measured parameters with the model predictions.

### 2.6. Core–Shell Aerogel Particles Production

Core–shell aerogel particles, both unloaded and drug-loaded, were prepared using alcogel optimized formulation from the previous section, following the protocol from Section 2.3. Briefly, solutions of KGM and alginate were prepared with concentrations of 0.68% *w/v* and 1.11% *w/v* of alginate, respectively. The prilling system (Encapsulator Unit Var J1, Nisco Engineering AG, Zurich, Switzerland) was equipped with the optimal coaxial nozzle configuration (C, 0.8/0.15 mm). The droplets were formed by a 10 cm drop height and crosslinked in a 96% ethanol solution containing 0.26 M calcium chloride under stirring at 250 rpm. An airflow rate of 1.75 L/min was used to atomize the polymeric dispersions from the alginate flask pressurized at 0.23 bar and from the KGM flask pressurized at 0.92 bar. The resulting particles were aged in the crosslinking bath for 5 min, filtered, and transferred to absolute ethanol (24 h) before the drying step.

Samples were dried in a 400 mL high-pressure autoclave (Eurotechnica, Bargteheide, Germany) at 40 °C and 120 bar. CO_2_ was introduced at a flow rate of 15 g/min for the first hour, followed by a reduced flow rate of 10 g/min for the remaining 2.5 h. After supercritical drying, the particles were collected and stored at 20 °C in dry atmosphere. The preparation process is illustrated in Figure 1A.

Drug-loaded particles were prepared by dispersing the drug into the alginate solution (1.11% *w/v*) under continuous stirring. Van (500 mg) was added to 50 mL of alginate solution and DX (250 mg) to 25 mL, both achieving a final drug concentration of 10 mg/mL. The alginate solution was stirred at 500 rpm during prilling to maintain a homogeneous DX suspension.

The different aerogel formulations were produced in triplicate and designated as follows: unloaded core–shell aerogels (Blank CS), DX-loaded core–shell aerogels (DX CS), and Van-loaded core–shell aerogels (Van CS).

### 2.7. Characterization of Aerogel Formulations

#### 2.7.1. Morphological Characterization

The particle size (Feret diameter) and circularity of the aerogel formulations were evaluated following the methodology in Section 2.4.

#### 2.7.2. Structural Characterization

The microstructural characteristics of the aerogel formulations were evaluated using nitrogen adsorption–desorption analysis (ASAP2000, Micromeritics Inc., Norcross, GA, USA). Aerogel samples (100 mg) were degassed under vacuum conditions (<1 mPa) at 50 °C for 24 h. The specific surface area was calculated using the BET method, while the pore diameter was determined using the BJH method [29,30].

Scanning electron microscopy (FESEM Ultra-plus, Zeiss, Oberkochen, Germany) was used to examine the surface and porous structure (core and shell sections) of the aerogels. Iridium was applied as a sputtered coating to enhance contrast, enabling higher magnification and clearer images.

#### 2.7.3. Drug Loading and Encapsulation Efficiency Characterization

The drug loading of Van CS aerogels was evaluated in 20 mg aliquots, which were immersed in 5 mL of distilled water and sonicated for 30 min to release the drug. A 1 mL sample of the release medium was filtered through a PTFE hydrophilic syringe filter (13 mm, 0.2 µm pore size) to minimize absorbance interference from alginate chains. Vancomycin was quantified spectrophotometrically in the filtrate with an Agilent 8453 (G1103A) UV/Vis equipment (Agilent Technologies, Waldbronn, Germany) at a wavelength of 282 nm using a previously validated calibration curve (*R*^2^ = 0.998) in the concentration range of 25–250 µg/mL.

The drug loading of DX CS aerogels was evaluated using a similar protocol. DX in the filtrate was quantified using High-Performance Liquid Chromatography using a JASCO system (AS-4140 autosampler, PU-4180 pump, LC-NetII/ADC interface box, CO-4060 column oven, MD-4010 photodiode array detector; JASCO, Tokyo, Japan), operated with ChromNAV software v2 following a previously established method, establishing a calibration curve (*R*^2^ = 0.999) using a wavelength of 242 nm, with a concentration range of 0.025–50 μg/mL [31]. The column used was a Symmetry^®^ C_18_ 5 µm, 3.9 × 150 mm (Waters, Milford, USA), and the mobile phase consisted of water/acetonitrile (67:33, *v/v*) at a flow of 1 mL/min operating in an isocratic regime. All experiments were carried out in triplicate.

Drug loading (*DL*, in %) and encapsulation efficiency (*EE*, in %) were calculated according to Equations (2) and (3), respectively:

(2) $$DL=\frac{m\;(drug\;in\;aerogels)}{m\;(loaded\;aerogels)}\times 100$$

(3) $$EE=\frac{m\;(drug\;in\;aerogels)}{m\;(drug\;dropped)}\times 100$$

*EE* was also estimated using an indirect method during alcogel preparation. This involved studying the mass balance of the drugs by determining their amount in the crosslinking and solvent exchange solutions and inferring the residual drug in the particles.

#### 2.7.4. In Vitro Drug Release Characterization

Drug release profiles from aerogel formulations were carried out in neutral and acidic solutions using PBS (pH 7.4) [32] and HCl 0.1 M (pH 1) [33] as dissolution media.

Van release profiles were obtained using 40 mg aliquots of Van CS aerogels in 15 mL of release medium. Tests were performed in triplicate. Beakers were placed in an orbital shaker (Unimax 1010, Heidolph, Schwabach, Germany) set at 90 rpm and thermostatized at 37 °C (Incubator 1000, Heidolph, Schwabach, Germany). Samples (1 mL) were collected at preset times (5, 10, 15, 20, 25, 30, 40, 50, 60, 90, and 120 min) using a syringe, filtered through a hydrophilic PTFE syringe filter (13 mm, 0.2 µm pore size), and analyzed spectrophotometrically as described in Section 2.7.3. After each sampling, an equal volume of fresh medium was added.

DX release was assessed using 60 mg aliquots of DX CS aerogel in 15 mL of release medium. Tests were performed in triplicate. The procedure was carried out with identical setup to the Van release test. Samples (1 mL or 0.9 mL for neutral and acidic medium, respectively) were collected at preset times (10, 20, 30, 45, 60, 120, 240, 480, and 1440 min) using a syringe and filtered through a hydrophilic PTFE syringe filter (13 mm, 0.2 µm pore size). After each sampling, the same volume of fresh medium was added. DX was determined by HPLC as described in Section 2.7.3. To prevent chromatographic column degradation, acidic samples (pH 1) were diluted with 0.8 mL of NaOH 0.1 M solution to raise the pH above 2 before filtration.

### 2.8. Statistical Analysis

All data were expressed as mean ± standard deviation. The particle size distribution of the final alcogel and aerogel formulations were characterized by the Probit method [34]. Statistical descriptors of these distributions were calculated using GraphPad Prism v.8.0.2 (Boston, MA, USA).

## 3. Results and Discussion

### 3.1. Preliminary Considerations for the Definition of the Space Design

This study successfully produced CS alcogel particles using coaxial prilling within a range of compositions and process parameters. In the prilling process, droplet formation is influenced by nozzle diameter, pressure, and dispersion concentration [35,36]. Insufficient flow rates hinder particle formation, while excessive flow rates lead to filament formation rather than droplet formation, impacting the quality. Therefore, the definition of appropriate limits is essential to ensure reproducibility and efficient results. These general principles are more challenging when using coaxial nozzles, as they necessitate precise control of two solution flow rates simultaneously [22]. In this study, nozzle configuration A (smallest space) required higher pressures and lower solution concentrations, whereas nozzle configuration C (larger space) allowed higher flow rates and pressures with lower solution concentrations. Maximum solution concentrations of 0.7% *w/v* KGM and 1.25% *w/v* Alg were established, with pressures ranging from 0.2 to 1.2 bar, depending on the nozzle configuration. Preliminary studies were conducted to determine these limits (Section 2.3). These tests identified the conditions that promoted consistent droplet formation and particle production across nozzle configurations. By defining compatible variable ranges (Table S1), the study minimized the risk of inadequate flow rates and ensured a more robust and efficient experimental design to successfully produce alcogel particles for subsequent characterization.

### 3.2. Modelling and Optimization of Core–Shell Alcogel Formulations Through Artificial Intelligence Techniques

#### 3.2.1. Neurofuzzy Logic (NFL) Modelling

Table 2 shows the characterization parameters for the prilling process and CS alcogel particles in the different formulations. Despite the preliminary studies of Section 3.1, trials of formulations 4, 7, 10, 19, and 22 failed due to polysaccharide solutions blockage in the nozzles. This was related to inadequate solution viscosity, nozzle size, and applied pressure, hindering the overcoming of capillary forces [13,22,37]. Finally, formulation 15 produced a continuous filament instead of particles due to high solution flow and insufficient airflow and was also discarded.

The results revealed a broad range of particle sizes (1.1 to 3.0 mm) and shapes across the different formulations, ranging from large, irregular forms (e.g., formulations 3 and 17) to smaller and more spherical particles (e.g., formulations 1 and 20). Coating thickness also exhibited significant variations, from formulations that had no coating (e.g., formulations 13 and 21) to shells of up to 0.35–0.40 mm (e.g., formulations 14 and 23).

Despite the limited number of formulations tested, FormRules^®^ succeeded in modelling the relationship between inputs and outputs, as demonstrated by the model’s predictability and accuracy values (Table 3). The ASMOD algorithm in FormRules^®^ generated interpretable multivariate models by identifying input-output relationships, further simplified by IF…THEN rules (Tables S2–S6) [17,22]. The principal submodel (Table 3, in bold) explains the majority of the output variability [27,38]. All models exhibit strong predictability (*R*^2^ > 73%), with accuracy (α < 0.05) observed for all outputs except for Feret diameter, attributed to model complexity and limited degrees of freedom.

The prilling capability score model revealed that nozzle blockage is highly dependent on its configuration. Nozzle configuration A (i.e., with shorter diameter) was more susceptible to blockage compared to nozzle configurations B and C, which allowed for smoother extrusion. This aligns with increased flow resistance in smaller nozzles, particularly for viscous solutions [39,40], where thicker solutions (from higher polymer concentration) and smaller nozzles would usually need higher pressures to make droplets [13,14].

Submodels identified Alg concentration and P_outer_ as critical parameters, with nozzle configuration being the most influential factor in preventing nozzle blockage and ensuring successful particle production. Submodel 2 (Table S2) confirmed that higher Alg concentration decreased prilling capability score. For KGM solutions, moderate P_outer_ improved the prilling capability score, but high pressures decreased it (Table S2, submodel 3). This counter-intuitive trend may be explained by frequency sweep studies, which show increased viscosity and elastic behaviour at high deformation forces when the storage moduli surpassed the elastic moduli [41,42].

The accuracy of the CS particle size model was limited and should be considered with caution. It suggests that particle size is mainly influenced by the interaction between nozzle configuration and P_outer_, together with KGM concentration and P_inner_, and airflow rate (Table S3). These findings are consistent with previous research works on prilling and extrusion processes, which link particle size with nozzle diameter and droplet breakup mechanisms [11,13,37,40,41,43]. According to submodel 3, the size of the particle (Table S3 at Supplementary Materials section) can be increased by reducing nozzle airflow rates. Although previous studies [13] indicated a notable decrease in particle size with increased airflow rate, submodel 3 predictions showed that this effect was limited, potentially due to the complexity of the model and the combined effect of other factors. Conversely, increased solution flow rates and longer nozzle diameters were reported to increase particle size [11,37,43]. According to the model, nozzle configuration B and P_outer_ effectively controlled particle size, with higher pressures yielding larger particles, and lower pressures yielding smaller particles. Nozzle configuration A consistently produced small particles, while nozzle configuration C produced larger particles at lower pressures. Finally, low KGM and Alg concentrations promoted larger particle formation, likely due to the reduced solution viscosity.

According to the model, the variability in circularity of the core–shell particles is significantly determined by the interaction of the nozzle configuration and P_outer_, together with the single effect of P_inner_, and the single effect of P_outer_ (Table 3 and Table S4).

The circularity of prilled particles is known to be dependent on several factors, including the viscosity of the extruded material, the surface tension between the droplets and the surrounding medium (first air, second liquid), and nozzle-to-crosslinking bath distance [44,45,46]. In this study, the models successfully captured established behaviours related to these variables (Table 3 and Table S4). Specifically, nozzle configuration B, with higher annular gap (0.65 mm), produced the most circular particles at low KGM pressure. Conversely, high KGM pressure in this nozzle configuration resulted in the least circular particles, likely due to excessive flow rates overcoming the available space. This suggests that while greater space facilitates circularity, high pressure can counteract this benefit. Configuration A (0.35 mm) and C (0.45 mm) provided less annular gap, impacting particle shape.

Furthermore, Alg (P_inner_) and KGM (P_outer_) pressures significantly affected particle circularity, according to experimental observations (Submodels 2 and 3 in Table S4). Higher Alg pressures increased circularity, while higher KGM pressures decreased it. This effect may be linked to the fixed nozzle-to-bath distance of 10 cm, which is more suited for Alg ionotropic gelation than precipitation gelation as KGM or chitosan. Alg gelation typically requires distances around 10–15 cm for high sphericity, whereas gelation by precipitation necessitates longer distances [47,48,49]. On this study, the distance nozzle-to-bath was 10 cm, reducing the circularity of particles when higher extrusion pressures of KGM solutions are used and thus making the nozzle-to-bath distance more critical.

The centred core score model showed that nozzle configuration and the balance between P_inner_ and P_outer_ also impacted the core positioning significantly (Table 3 and Table S5). Nozzle configuration A consistently failed to produce centred core particles, while nozzle configurations B and C, particularly at low P_inner_ and medium-high P_outer_, yielded the most centred core particles (Table 3 and Table S5). Previous coaxial prilling studies suggest that core centring depends on several factors: increased inner-to-outer viscosity ratio (µ_inner_/µ_outer_) with constant pump flow rates [50], higher inner pump flow rates with higher outer concentrations using 450/900 µm nozzles [51], and higher outer concentrations with 400/600 µm nozzles and constant pump flow rates. Our results aligned with the influence of high outer solution pump flow rates but differed regarding inner pump flow rates. Previous works with 450/900 µm nozzles (similar to nozzle configuration C, with 350/800 µm) showed that high inner pump flow rates yielded centred cores. Our model did not support this trend, but a direct comparison between both works is regarded as limited due to variations in equipment and materials. Firstly, KGM was used in the outer layer, which exhibits less uniform gelation compared to polymers like alginate gelified with calcium chloride. Therefore, a smaller amount of inner solution might be necessary to ensure complete coating. Secondly, the shrinkage of KGM during precipitation/gelation could be greater than that of alginate, affecting core centring.

Further considerations include the volume of coating solution dropped during the prilling. Nozzle configurations B and C, with their larger available space, increase the amount of dripped solution, potentially enhancing homogeneous core coating. Similarly, decreasing coating solution concentration facilitates greater dripping of the solution, achieving a comparable effect [50].

According to the model, coating thickness, defined as the distance from the surfaceto the core of a particle, is primarily influenced by KGM concentration and nozzle airflow (Table 3 and Table S6). At low KGM concentrations, increased airflow rate resulted in reduced coating thickness. This suggest that higher airflow rates disrupt droplets formation, leading to less KGM deposition per drop. Similar trends were reported in vibration-assisted coaxial prilling, where higher frequencies lead to thinner coatings [22]. Furthermore, the balance between polymer concentration and solution pressures influenced coating thickness by determining the KGM/Alg volume ratio in the beads, in agreement with other studies [11,50]. Extreme KGM/Alg volume ratios resulted in uncoated particles, either pure KGM or pure Alg, as reflected in submodels 1 and 3 (Table 3 and Table S6). KGM concentration was important within the pressure and concentration ranges selected for the design space of the study.

FormRules models effectively captured and expanded upon existing knowledge of core–shell prilling. The multivariable analysis provided a unique perspective, revealing key insights into KGM behaviour and airflow rate effects. By simultaneously evaluating critical factors like prilling capability, particle size, and core centering, the models highlighted the importance of nozzle configuration, nozzle airflow, and inner or outer pressures. Furthermore, they offered explanations for the observed discrepancies, demonstrating the power of FormRules in comprehensively understanding complex prilling processes.

#### 3.2.2. Optimization by Artificial Neural Networks and Genetic Algorithms

The complex interplay of the variables studied often shows contradictory effects on core–shell particle characteristics. For example, while a decreasing airflow rate increases particle size, it simultaneously reduces coating thickness, creating a direct conflict when attempting to optimize both parameters at the same time. In those cases, a robust optimization strategy is essential. INForm^®^, a hybrid software that combines ANNs and GAs to manage high-dimensional design spaces, efficiently searches for optimal solutions by mimicking natural selection. By leveraging the predictive power of ANNs and the optimization capabilities of GAs, INForm^®^ modelled the database (training and test data *R*^2^ > 0.74) and can handle multiple objectives and constraints simultaneously, allowing for the fine-tuning of particle size, shape, centred core, and coating thickness, despite conflicting variable influences. By iteratively refining potential solutions through selection, crossover, and mutation, GAs can identify the optimal combination of variables, leading to the production of core–shell particles with precisely tailored properties.

Although the models exhibited limited accuracy due to the dataset size, INForm^®^ effectively utilized defined desirability functions (Table 4) to achieve a 100% desirable solution (Table 4), meaning that the software was able to attain a solution where all the pre-defined desirability conditions were met. This suggests a prilling process of the minimum risk of blockage which yields homogeneous batches of spherical particles (2–3 mm diameter) with centred cores and maximum coating thickness.

**Figure 2:**
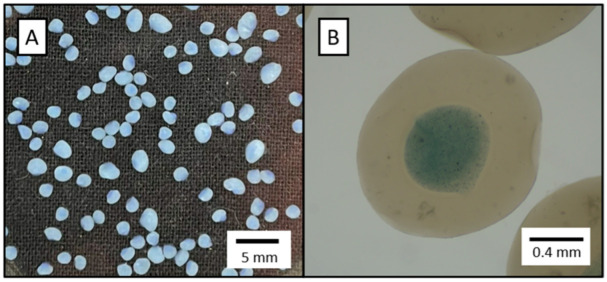
Optimized alcogel morphology: (**A**) digital camera image; (**B**) microscope image. Blue colourant was used to highlight the core-coating differentiation.

INForm^®^ selected the following parameters for the optimal formulation: nozzle configuration C, 0.68% *w/v* KGM concentration, 1.11% *w/v* alginate concentration, P_outer_ = 0.92 bar, P_inner_ = 0.23 bar, and airflow rate of 1.75 L/min. Model validation was conducted by experimentally producing the optimized formulation. The experimental results (Table 5) were in close agreement with those predicted by the model. Figure 2 displays the morphological characteristics of the resulting alcogels.

### 3.3. Characterization of Core–Shell Aerogel Particles

#### 3.3.1. Morphology, Particle Size Distribution, and Textural Properties of Core–Shell Aerogels

**Figure 3:**
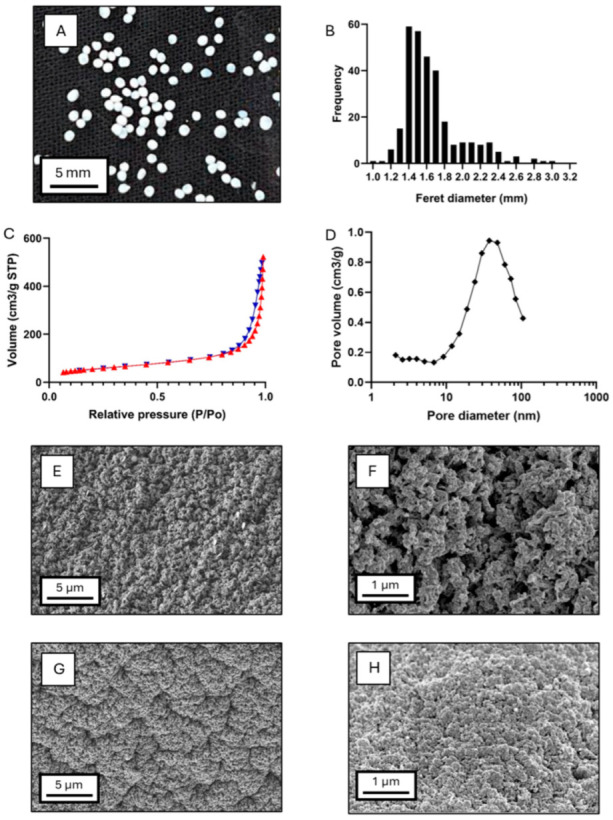
Characterization of the core–shell aerogels obtained from the optimized alcogel formulation. (**A**) Digital camera image of the particles, (**B**) particle size distribution (by static image analysis), (**C**) nitrogen adsorption (red triangles) and desorption (blue triangles) isotherms, (**D**) pore size distribution (BJH method). SEM images of the nanostructure of (**E**,**F**) the KGM coating and (**G**,**H**) the alginate core.

Aerogels produced by supercritical drying from the optimized alcogels were characterized in terms of their particle size, morphology, and microstructure (Figure 3A,B). Aerogel particle sizes ranged from 0.90 and 3.30 mm, with an average diameter of 1.57 mm, a geometric deviation of 1.16, and Q_25_ = 1.40 mm, Q_50_ = 1.51 mm, and Q_75_ = 1.70 mm. Probit analysis confirmed a log-normal size distribution (R^2^_Log-normal_ = 0.967) [34]. This trend was also observed in the precursor alcogel particles, which exhibited Feret diameters between 1.30 and 3.60 mm, an average size of 1.77 mm, a geometric deviation of 1.15, and Q_25_ = 1.55 mm, Q_50_ = 1.68 mm, and Q_75_ = 1.93 mm. Their distribution also fitted to a log-normal distribution (R^2^_Log-normal_ = 0.887). Therefore, the log-normal size distribution was preserved during supercritical drying, despite an overall particle shrinkage, which is characteristic of the supercritical drying process [48,52].

Nitrogen adsorption–desorption analysis was used to analyze the mesoporosity of the aerogels and revealed a material with a high specific surface area of 201 ± 10 m^2^/g, an overall specific pore volume of 0.78 ± 0.04 cm^3^/g, and a mean pore diameter of 15.4 ± 0.8 nm. The type IV isotherm (Figure 3C) with hysteresis loop type H1 indicates capillary condensation in the mesoporous structure of the aerogel, suggesting the formation of agglomerated chains with rigid bounds and relatively uniform pores [29,53]. Pore size distribution (Figure 3D) showed a dominant pore family centred at 40 nm and a small region of 2–5 nm mesopores. Due to the core–shell composition with two polysaccharides, individual material contributions to textural properties are unclear. Comparisons with other aerogels should be cautious, as glucomannan aerogels from supercritical drying were not previously produced so far.

Macropore characterization was carried out by SEM microscopy. Images (Figure 3E–H) showed distinct porous structures between the outer and the inner parts of the core–shell aerogels. Both regions exhibited dense networks, likely due to hydrophobic interactions between the polysaccharide chains, induced during ethanolic crosslinking [53]. Macropore differences were observed between materials, with larger macropores in the KGM shell than in the Alg core. This confirms the presence of both meso- and macropores in the core–shell particles. Again, the comparison with other polysaccharide aerogels must be made cautiously, as variations in polysaccharide concentration, crosslinking method, solvent exchange protocol, and supercritical drying conditions significantly impact the aerogel matrix structure [6,30,53].

#### 3.3.2. Drug Loading, Entrapment Yield, and Drug Release Studies: Hydrophilic Vs. Hydrophobic Drugs

Core–shell aerogels were loaded with Van and DX during gelation. Van was dissolved and DX was dispersed in the inner solution of the core–shell particles. Gelation occurred upon contact with a calcium ion-containing ethanol bath, resulting in immediate particle formation [11]. Specifically, the alginate is crosslinked by a synergistic effect of the calcium cations and the precipitation induced in contact with the ethanol. For deacetylated KGM, the contact with ethanol induces a fast gelation produced by the precipitation of the KGM chains. To promote a suitable gelation, it is necessary the previous deacetylation process. Van, being poorly soluble in ethanol, was expected to experience reduced mass losses, further aided by the coating barrier effect. Conversely, DX, with higher ethanol solubility, was expected to exhibit higher losses, although coating could mitigate this to some extent [10,54]. Due to the low solubility of both drugs in scCO_2_, minimal losses were expected during supercritical drying [55,56].

Van-loaded core–shell aerogels exhibited a DL of 11.79 ± 2.76% and an EY of 17.30 ± 3.34%. These values were consistent with previous studies on alginate or chitosan prepared by prilling and loaded during the gelation process [48,52]. However, a loss of Van of 44.65 ± 8.76% was observed during alcogel preparation, with an additional 40% loss during supercritical drying, potentially due to EtOH-CO_2_ supercritical mixture formation in the autoclave.

For DX-loaded core–shell aerogels, DL was 0.032 ± 0.001%, and EY was 0.182 ± 0.003%. A significant loss of DX (98.41 ± 8.96%) occurred during crosslinking in ethanol and solvent exchange processes, likely due to the high ethanol solubility of DX and the low retention capacity of the polysaccharide gels [10,54]. Given the substantial drug loss during the current procedure, a refined drug loading strategy, incorporating reduced solvent exchange exposure, or modified crosslinking techniques, is crucial for achieving acceptable drug-loading levels.

The selection of drugs for aerogel formulations, along with the corresponding loading mechanisms, is critical to minimize drug loss [4]. In the preparation protocol used in this study, drugs can be loaded either during the preparation of the polymer solutions or in the final stage of the solvent exchange process. An alternative approach involves post-supercritical drying loading through a technique known as impregnation [3].

To minimize drug loss during aerogel preparation, incorporating the drug at the stage in which its solubility is highest (whether in aqueous-based solutions, ethanol, or supercritical CO_2_) is recommended [4]. In the case of DX, loading during the solvent exchange step is recommended due to its higher solubility in ethanol. However, this method may result in drug accumulation in the outer shell of the particles, potentially compromising their intended function. In this work, the production of core–shell particles by loading the drug during the preparation of Alg solution affects the DL of the final aerogels, particularly for DX-loaded aerogels, supposing an important limitation.

To enhance drug loading (DL) in future core–shell formulations, reducing the volume of solvents used during gelation and solvent exchange could be a promising strategy. Nonetheless, this approach presents several technical challenges, including achieving efficient crosslinking with limited volumes of crosslinking agents and ensuring complete solvent exchange with ethanol while maintaining the porous structure of the gels.

**Figure 4:**
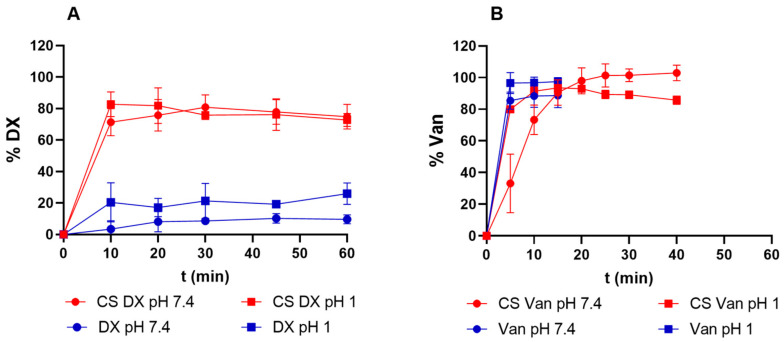
Drug release profiles from core–shell aerogel particles compared with dissolution profiles of crystalline drug. (**A**) DX release from core–shell aerogels (red) and crystalline DX (blue) at pH 1 (squares) and pH 7.4 (circles), (**B**) Van release profile from core–shell aerogels (red) and crystalline Van (blue) at pH 1 (squares) and 7.4 (circles).

Both Van and DX exhibited a burst release from the core–shell aerogels formulations, within 1 h in both PBS pH 7.4 and 0.1 M HCl (pH 1) solution media (Figure 4. However, the drug release profiles from aerogels differed from the dissolution profiles of pure drugs.

Pure DX base demonstrated limited dissolution, 30% in HCl 0.1 M and 15% in PBS within 2 h, progressing to complete dissolution in acidic medium and 80% in PBS after 24 h (Figure 4A). In contrast, DX-loaded aerogels released approximately 80% of the drug within 10 min in both solutions. This rapid release can be attributed to the high surface area and the mesoporosity of aerogels, which may facilitate molecular dispersion of DX through the structure against the free DX [57,58].

Pure Van dissolved rapidly in both solutions (less than 5 min, Figure 4B). Conversely, Van-loaded aerogels exhibited a medium dependent release profile (Figure 4B). In acidic medium (pH 1), 80% of the Van was released within 5 min, while complete release took 20 min in PBS medium at pH 7.4. In contrast to our findings, previous studies on core–shell particles prepared by prilling and loaded with hydrophilic drugs demonstrated sustained release patterns [10,11,54]. Successful sustained release was achieved using both ionotropic crosslinking with Ca^2+^ [10,54] and coagulation crosslinking combined with Ca^2+^ [11] for small molecules. Also, for peptidic drugs like insulin, core–shell alginate aerogels crosslinked with Ca^2+^ retained 65% of drug in acidic conditions, whereas the total drug release took place in 120 min in Simulated Colonic Fluid medium [12]. In our work, a deacetylated KGM solution was used for the coating, differing from the previously reported works. This difference may have resulted in a coating with reduced drug retention capabilities or a less effective crosslinking network, explaining the observed discrepancy in the release profiles. To address these challenges, future studies could explore alternative strategies such as modifying formulation components and crosslinking strategies.

Regarding the pH conditions used in the drug release experiments, they were selected based on their relevance to oral drug delivery studies, as they simulate the physiological environments of the gastrointestinal tract (acidic in the stomach and neutral in the intestine) to evaluate the influence of pH on drug release profiles.

## 4. Conclusions

This study successfully designed and optimized novel polysaccharide core–shell aerogel particles composed of an alginate core and a KGM shell using an air-assisted coaxial prilling technique followed by scCO_2_ drying. This research represents the first reported production of aerogels with KGM, demonstrating its feasibility as a functional material in aerogel formulations. The resulting aerogel particles exhibited desirable structural characteristics, including high specific surface area and dual macro- and mesoporosity.

The AI techniques used proved invaluable for the effective modelling and optimization of the prilling process, efficiently identifying nozzle configuration, flow rates, and pressures as critical parameters. This AI-driven approach enabled the precise control and manipulation of the hydrogel properties, allowing us to achieve a 100% desirable formulation after defining the appropriate desirability functions and applying genetic algorithms, even within the inherent complexity and often opposing effects of numerous process variables or their interactions. This demonstrates AI’s ability to navigate multivariate design spaces and achieve optimal outcomes in the development of complex drug delivery systems.

Two limitations of this study were found. The first are the unexpected drug release patterns of Van and DX from the aerogels, which contradicted the sustained release patterns typically observed using similar prilling techniques with Alg or other polysaccharides. This discrepancy was attributed to the use of the deacetylated KGM coating, which exhibited reduced drug retention capabilities, or a less effective crosslinking compared to previously reported coatings. Second, the formulations present low DL values, specially for DX-loaded aerogels. This is primarily attributed to the multistep process required to obtain the final aerogel form.

This work provides a foundational understanding of KGM’s potential in aerogel formulations and highlights the need for further improvement of the coating composition, crosslinking methods, and reducing solvent volumes during processing to achieve tuneable drug release profiles and higher DL values. The successful application of AI modelling opens the door to future research focused on tailoring KGM-based aerogels for specific therapeutic applications.

## Figures and Tables

**Table 1:** Experimental design for six variables established by DataForm^®^ v.3.1 to produce the core–shell alcogel formulations (F).

Sample	NC	[KGM] (% *w/v*)	[Alg] (% *w/v*)	P_outer_ (bar)	P_inner_ (bar)	Airflow (L/min)
1	B	0.7	0.75	0.4	0.6	2.20
2	C	0.6	1.25	0.8	1.0	2.65
3	A	0.7	0.75	1.2	0.2	1.75
4	A	0.6	1.25	0.8	0.2	1.75
5	B	0.7	0.75	0.4	0.6	2.65
6	C	0.6	1.25	1.2	1.0	2.20
7	A	0.6	1.25	0.8	0.2	2.65
8	B	0.7	0.75	0.4	0.8	1.75
9	C	0.7	0.75	1.2	1.0	2.20
10	A	0.6	1.25	0.4	0.6	1.75
11	B	0.7	1.25	1.2	1.0	2.20
12	C	0.6	0.75	0.8	0.2	2.65
13	C	0.7	1.25	0.4	1.0	2.65
14	A	0.6	0.75	0.8	0.2	2.20
15	B	0.6	0.75	1.2	0.6	1.75
16	A	0.7	1.25	0.8	0.6	1.75
17	B	0.7	0.75	1.2	0.8	2.20
18	C	0.6	1.25	0.8	0.6	2.65
19	A	0.6	1.25	1.2	0.2	2.20
20	B	0.7	0.75	0.8	0.8	2.65
21	C	0.7	0.75	0.4	1.0	1.75
22	B	0.6	1.25	0.4	0.2	2.65
23	A	0.6	0.75	1.2	1.0	2.20
24	C	0.7	1.25	0.8	0.6	1.75

**Table 2:** Characteristics of 24 alcogel formulations (cf. Table 1 for experimental conditions). Reported results are prilling capability score (qualitative assessment for nozzle blockage), mean Feret diameter (particle size), circularity (particle shape), centred core score (core positioning), and coating thickness.

Sample	Prilling Capability Score	Mean Feret Diameter (mm)	Circularity	Centred Core Score	Coating Thickness (mm)
1	2	1.474	0.952	4	0.298
2	2	1.324	0.918	3	0.074
3	0	2.417	0.738	0	0
4	*	*	*	*	*
5	1	1.384	0.929	1	0.306
6	1	1.769	0.905	3	0.307
7	*	*	*	*	*
8	2	1.477	0.946	1	0.323
9	2	1.440	0.882	3	0.091
10	*	*	*	*	*
11	1	2.408	0.742	0	0
12	2	1.681	0.825	5	0.170
13	2	1.428	0.891	0	0
14	2	1.599	0.823	1	0.395
15	*	*	*	*	*
16	0	1.610	0.915	0	0
17	2	3.022	0.642	0	0
18	2	1.216	0.902	3	0.089
19	*	*	*	*	*
20	2	1.107	0.944	2	0.254
21	2	1.486	0.924	0	0
22	*	*	*	*	*
23	0	1.909	0.829	2	0.343
24	2	1.707	0.916	4	0.139

Asterisk symbol indicates failed formulation without data available.

**Table 3:** Model results and quality parameters. Inputs selected by FormRules^®^ to explain the variability of the different outputs. The most relevant submodels for each output are bolded.

Output	Submodels	Inputs from NFL Submodels	*R* ^2^	Calculated F Value	d.f. (v1, v2)	Critical F α < 0.05
Prilling capability score	**1**	**NC**	73.88	7.07	6, 15	2.79
	2	[Alg]				
	3	P_outer_				
Mean Feret diameter	**1**	**NC ×** **P_outer_**	93.12	1.80	15, 2	19.45
	2	[KGM] × P_inner_				
	3	Airflow				
Circularity	**1**	**NC ×** **P_outer_**	92.07	10.31	9, 8	3.38
	2	P_inner_				
	3	P_outer_				
Centred core score	**1**	**NC ×** **P_inner_**	75.25	4.17	8, 11	2.94
	2	P_outer_				
Coating thickness	1	[Alg]	89.72	7.85	10, 9	3.14
	**2**	**[KGM] ×** **Airflow**				
	3	P_outer_ × P_inner_				

d.f. = degrees of freedom.

**Table 4:** Desirability function definitions for optimization, showing Min. (minimum), Max. (maximum), and intermediate (Mid1., Mid2) values that define the transition point (up or tent) for each parameter desirability function, along with assigned weights used in genetic algorithm optimization.

Property	Weight	Function	Min	Mid1	Mid2	Max
Prilling capability score	10	UP	0	1.5	1.6	2
Mean Feret Diameter	5	TENT	1.107	2	2.5	3.022
Circularity	1	UP	0.642	0.7985	0.7985	0.955
Centred core score	9	UP	0	4	4.1	5
Coating thickness	8	UP	0.074	0.2345	0.2345	0.395

**Table 5:** Predicted vs. experimental results for optimized alcogel formulation. INForm^®^ predicted values (no SD) and experimental values (average ± SD, *n* = 300 particles).

Property	Prilling Capability Score	Mean Feret Diameter (mm)	Circularity	Centred Core Score	Coating Thickness (mm)
Theoretical	2	2.10	0.86	4	0.28
Experimental	2 ± 0	1.83 ± 0.43	0.89 ± 0.13	4 ± 0	0.32 ± 0.15

## Data Availability

Data will be made available on request.

## Supplementary Materials

The following supporting information can be downloaded at https://www.mdpi.com/article/10.3390/polym17141919/s1, Table S1: Qualitative observations to establish the limits of the variables for the experimental design. Table S2: Rules for the prilling capability score; Table S3: Rules for the mean Feret diameter; Table S4: Rules for circularity; Table S5: Rules for centred core; Table S6: Rules for coating thickness.
