# Supplementary material for: Novel Core–Shell Aerogel Formulation for Drug Delivery Based on Alginate and Konjac Glucomannan: Rational Design Using Artificial Intelligence Tools
Source: Polymers (Basel). 2025 Jul 11;17(14):1919. doi: 10.3390/polym17141919 (PMC12298074; doi:10.3390/polym17141919)
Supplement: Supplementary file 1 [file polymers-17-01919-s001.zip › polymers-3715960-supplementary.pdf]

Supplementary information

Novel Core–Shell Aerogel Formulation for Drug Delivery Based on Alginate and Konjac Glucomannan: Rational Design using Artificial Intelligence Tools

Carlos Illanes-Bordomás, Mariana Landin*, Carlos A. García-González*

AerogelsLab, I+D Farma Group (GI-1645), Department of Pharmacology, Pharmacy and Pharmaceutical Technology, iMATUS and Health Research Institute of Santiago de Compostela (IDIS), Universidade de Santiago de Compostela, E-15782 Santiago de Compostela, Spain; carlosjavier.illanes@rai.usc.es (C.I.B.), m.landin@usc.es (M.L.), carlos.garcia@usc.es (C.-A.G.-G.).

* Correspondence: m.landin@usc.es (M.L.), carlos.garcia@usc.es (C.A.G.-G.)

Table S1: Qualitative observations to establish the limits of the variables for the experimental design.

Variable	Limit	Observations
[KGM]	Lower: 0.6% w/v	Below this limit, particles did not exhibit rigid crosslinking
	Upper: 0.7% w/v	Above this concentration, the solution cannot be dropped through the smallest nozzle configuration due to clogging or slow flow rates (Configuration A)
[Alg]	Lower: 0.75 % w/v	Below this limit, the solution flow rate was too fast for configuration B, sometimes forming a liquid jet
	Upper: 1.25 % w/v	The solution flow rate was too slow for configuration A
P_{outer}	Lower: 0.4 bar	It is not possible to drop the 0.7% w/v KGM solutions through nozzle configurations A and C
	Upper: 1.2 bar	The operational upper limit of the equipment is 1.5 bar
P_{inner}	Lower: 0.2 bar	The operational lower limit of the equipment is 0.1 bar
	Upper: 1.0 bar	The solution flow rate became too high (forming liquid jet) in configuration C
Airflow	Lower: 1.75 L/min	Particle sizes significantly increased at the tip of the nozzle
	Upper: 2.65 L/min	The particles break up at the tip of the nozzle and the dropping directions were sometimes not vertical

Table S2: Rules for the Prilling capability score

**Rules for Prilling capability score**			
	SubModel 1		
1	IF Nozzle configuration is B	THEN Prilling capability score is	HIGH (0.88)
2	IF Nozzle configuration is C	THEN Prilling capability score is	HIGH (1.00)
3	IF Nozzle configuration is A	THEN Prilling capability score is	LOW (1.00)
	SubModel 2		
4	IF [Alg] is LOW	THEN Prilling capability score is	HIGH (1.00)
5	IF [Alg] is HIGH	THEN Prilling capability score is	LOW (0.83)
	SubModel 3		
6	IF P_{outer} is LOW	THEN Prilling capability score is	LOW (0.81)
7	IF P_{outer} is MID	THEN Prilling capability score is	HIGH (1.00)
8	IF P_{outer} is HIGH	THEN Prilling capability score is	LOW (0.78)

Note: Blue colour is used to indicate the combination of inputs giving the highest value of the output and red colour is used to indicate the one giving the lowest value of the output

Table S3: Rules for the Mean Feret Diameter.

Rules for Mean Feret Diameter			
	SubModel 1		
1	IF P_{outer} is LOW AND Nozzle configuration is B	THEN Mean Feret Diameter is	LOW (1.00)
2	IF P_{outer} is LOW AND Nozzle configuration is C	THEN Mean Feret Diameter is	HIGH (0.71)
3	IF P_{outer} is LOW AND Nozzle configuration is A	THEN Mean Feret Diameter is	LOW (1.00)
4	IF P_{outer} is MID AND Nozzle configuration is B	THEN Mean Feret Diameter is	LOW (1.00)
5	IF P_{outer} is MID AND Nozzle configuration is C	THEN Mean Feret Diameter is	LOW (1.00)
6	IF P_{outer} is MID AND Nozzle configuration is A	THEN Mean Feret Diameter is	LOW (1.00)
7	IF P_{outer} is HIGH AND Nozzle configuration is B	THEN Mean Feret Diameter is	HIGH (1.00)
8	IF P_{outer} is HIGH AND Nozzle configuration is C	THEN Mean Feret Diameter is	LOW (0.58)
9	IF P_{outer} is HIGH AND Nozzle configuration is A	THEN Mean Feret Diameter is	LOW (0.54)
	SubModel 2		
10	IF P_{inner} is LOW AND [KGM] is LOW	THEN Mean Feret Diameter is	HIGH (0.83)
11	IF P_{inner} is LOW AND [KGM] is HIGH	THEN Mean Feret Diameter is	HIGH (0.75)
12	IF P_{inner} is MID AND [KGM] is LOW	THEN Mean Feret Diameter is	LOW (0.94)
13	IF P_{inner} is MID AND [KGM] is HIGH	THEN Mean Feret Diameter is	HIGH (0.86)
14	IF P_{inner} is HIGH AND [KGM] is LOW	THEN Mean Feret Diameter is	LOW (0.75)
15	IF P_{inner} is HIGH AND [KGM] is HIGH	THEN Mean Feret Diameter is	LOW (1.00)
	SubModel 3		
16	IF Airflow is LOW	THEN Mean Feret Diameter is	HIGH (0.92)
17	IF Airflow is HIGH	THEN Mean Feret Diameter is	HIGH (0.66)

Note: Blue colour is used to indicate the combination of inputs giving the highest value of the output and red colour is used to indicate the one giving the lowest value of the output

Table S4: Rules for Circularity.

Rules for Circularity			
	SubModel 1		
1	IF P_{outer} is LOW AND Nozzle configuration is B	THEN Circularity is	HIGH (1.00)
2	IF P_{outer} is LOW AND Nozzle configuration is C	THEN Circularity is	LOW (0.98)
3	IF P_{outer} is LOW AND Nozzle configuration is A	THEN Circularity is	LOW (0.63)
4	IF P_{outer} is HIGH AND Nozzle configuration is B	THEN Circularity is	LOW (1.00)
5	IF P_{outer} is HIGH AND Nozzle configuration is C	THEN Circularity is	HIGH (0.82)
6	IF P_{outer} is HIGH AND Nozzle configuration is A	THEN Circularity is	LOW (0.54)
	SubModel 2		
7	IF P_{inner} is LOW	THEN Circularity is	LOW (0.64)
8	IF P_{inner} is HIGH	THEN Circularity is	HIGH (1.00)
	SubModel 3		
9	IF P_{outer} is LOW	THEN Circularity is	HIGH (0.87)
10	IF P_{outer} is MID	THEN Circularity is	HIGH (1.00)
11	IF P_{outer} is HIGH	THEN Circularity is	LOW (1.00)

Note: Blue colour is used to indicate the combination of inputs giving the highest value of the output and red colour is used to indicate the one giving the lowest value of the output.

Table S5: Rules for Centred core.

Rules for Centred Core score			
	SubModel 1		
1	IF Nozzle configuration is B AND P_{inner} is LOW	THEN Centred core score is	HIGH (1.00)
2	IF Nozzle configuration is B AND P_{inner} is HIGH	THEN Centred core score is	LOW (1.00)
3	IF Nozzle configuration is C AND P_{inner} is LOW	THEN Centred core score is	HIGH (1.00)
4	IF Nozzle configuration is C AND P_{inner} is HIGH	THEN Centred core score is	LOW (0.85)
5	IF Nozzle configuration is A AND P_{inner} is LOW	THEN Centred core score is	LOW (1.00)
6	IF Nozzle configuration is A AND P_{inner} is HIGH	THEN Centred core score is	LOW (1.00)
	SubModel 2		
7	IF P_{outer} is LOW	THEN Centred core score is	LOW (0.88)
8	IF P_{outer} is MID	THEN Centred core score is	HIGH (0.87)
9	IF P_{outer} is HIGH	THEN Centred core score is	HIGH (0.82)

Note: Blue colour is used to indicate the combination of inputs giving the highest value of the output and red colour is used to indicate the one giving the lowest value of the output.

Table S6: Rules for coating thickness.

Rules For Coating Thickness			
	SubModel 1		
1	IF [Alg] is LOW	THEN coating thickness is	HIGH (1.00)
2	IF [Alg] is HIGH	THEN coating thickness is	LOW (0.57)
	SubModel 2		
3	IF [KGM] is LOW AND Airflow is LOW	THEN coating thickness is	HIGH (1.00)
4	IF [KGM] is LOW AND Airflow is HIGH	THEN coating thickness is	LOW (1.00)
5	IF [KGM] is HIGH AND Airflow is LOW	THEN coating thickness is	LOW (1.00)
6	IF [KGM] is HIGH AND Airflow is HIGH	THEN coating thickness is	LOW (1.00)
	SubModel 3		
7	IF P_{inner} is LOW AND P_{outer} is LOW	THEN coating thickness is	HIGH (1.00)
8	IF P_{inner} is LOW AND P_{outer} is HIGH	THEN coating thickness is	LOW (1.00)
9	IF P_{inner} is MID AND P_{outer} is LOW	THEN coating thickness is	HIGH (1.00)
10	IF P_{inner} is MID AND P_{outer} is HIGH	THEN coating thickness is	LOW (1.00)
11	IF P_{inner} is HIGH AND P_{outer} is LOW	THEN coating thickness is	LOW (1.00)
12	IF P_{inner} is HIGH AND P_{outer} is HIGH	THEN coating thickness is	LOW (0.78)

Note: Blue colour is used to indicate the combination of inputs giving the highest value of the output and red colour is used to indicate the one giving the lowest value of the output.
